# Plant and soil fungal but not soil bacterial communities are linked in long-term fertilized grassland

**DOI:** 10.1038/srep23680

**Published:** 2016-03-29

**Authors:** Noriko A. Cassman, Marcio F. A. Leite, Yao Pan, Mattias de Hollander, Johannes A. van Veen, Eiko E. Kuramae

**Affiliations:** 1 Netherlands Institute of Ecology NIOO-KNAW, Department of Microbial Ecology, Wageningen, 6708 PB, Netherlands; 2 Leiden University, Department of Biology, Leiden, 2311 EZ, Netherlands; 3 Maranhão State University, Department of Agroecology, São Luís Maranhão, 65055-000, Brazil

## Abstract

Inorganic fertilization and mowing alter soil factors with subsequent effects–direct and indirect - on above- and below-ground communities. We explored direct and indirect effects of long-term fertilization (N, P, NPK, Liming) and twice yearly mowing on the plant, bacterial and fungal communities and soil factors. We analyzed co-variation using 16S and 18S rRNA genes surveys, and plant frequency and edaphic factors across treatments. The plant and fungal communities were distinct in the NPK and L treatments, while the bacterial communities and soil factors were distinct in the N and L treatments. Plant community diversity and evenness had low diversity in the NPK and high diversity in the liming treatment, while the diversity and evenness of the bacterial and fungal communities did not differ across treatments, except of higher diversity and evenness in the liming treatment for the bacteria. We found significant co-structures between communities based on plant and fungal comparisons but not between plant and bacterial nor bacterial and fungal comparisons. Our results suggested that the plant and fungal communities are more tightly linked than either community with the bacterial community in fertilized soils. We found co-varying plant, bacterial and fungal taxa in different treatments that may indicate ecological interactions.

The plant-soil feedback drives primary productivity and is fundamental to terrestrial ecosystem functioning^1^. Plant-derived litter and rhizo-deposits present C resources to soil microbes, and microbial decomposition of plant litter and soil organic matter (SOM) releases available nutrients to plants^2^. Furthermore, the plant-soil feedback underlies plant and soil microbial species interactions, many mediated by nutrient quality and availability. For example, plant species select a subset of the soil microbial (bacterial and fungal) community around the roots and in the topsoil through the biochemical composition of rhizo-deposits and leaves^3^,^4^,^5^. Nutrient competition, especially for N^6^, plays an important role in structuring bacterial communities over time in cooperation with the overlying plant species^7^,^8^. Moreover, plant and microbial species may be linked through negative pathogenic interactions or positive symbiotic associations, for example those of plants with Arbuscular Mycorrhizal Fungi (AMF)^9^ or N-fixing bacteria^1^ to enhance plant nutrient uptake. Through micro-climatic change, such as modulation of soil pH and subsequent effects on soil nutrient status, plant species may also indirectly alter the composition of microbial communities^2^. Thus, above- and below-ground communities may have significant associations at the community level. While links between soil microbial community diversity and biomass, and plant productivity^1^,^2^,^10^ or soil C storage^8^ are known, links between above- and below-ground community compositions have not received as much attention.

Altered plant, bacterial and fungal species interactions may have consequences for ecosystem function. Long-term grassland management practices of adding nitrogen (N), potassium (K) and phosphate (P) intended to increase vegetation productivity affect the composition and diversity of the plant community and the composition of the soil bacterial community^11^,^12^; this management practice can be considered a disturbance on above- and below-ground community compositions^13^. Nutrient additions can affect community compositions i) directly, e.g. by favoring species functionally adapted to the nutrient inputs and over time selecting those species so that they increase in abundance, or indirectly; these indirect effects include iia) the indirect effect of nutrient additions on soil physicochemical status, which can drive community composition changes (e.g. nutrient additions alter micronutrient availability which can structure microbial community compositions), iib) an indirect effect of nutrient inputs on the biomass of other communities which alters the composition of the original community; for example, nutrient additions increase plant productivity, which provides more C resources, accelerating soil microbial growth, and iic) an indirect effect of nutrient inputs on the compositions of other communities inducing shifts in the original community, e.g. nutrient additions favor some plant species, which may also favor microbial symbionts of these plants.

Here, we explored the related effects of long-term nutrient addition on the composition, evenness and diversity of three grassland communities – plant and soil bacterial and fungal assemblages as measured by 16S and 18S rRNA gene sequence surveys. We examined the direct effect of nutrient addition on the plant, bacterial and fungal communities across the treatments. We hypothesized H1) that each treatment would affect plant, bacterial and fungal communities differently. To look at the indirect effect of nutrient additions through altering soil factors, we considered the co-variation in soil factor profiles and plant, bacterial and fungal communities. In addition, we included a treatment in which lime (L) was added to raise the pH of the soil to near-neutral values, consequently altering macro and micronutrients availability. We hypothesized H2) that each community – plant, bacterial and fungal – would be affected by the L treatment compared to the unfertilized treatment, confirming the indirect effect of nutrient additions through altering soil factors on each community. We examined the indirect effect of nutrients through changes in a different community, in which altering the composition or diversity of another communities alters the composition or diversity of the original community, by examining the co-variation in communities across the treatments. Because of the importance of the plant-soil feedback, we hypothesized H3) that each community would co-vary across the treatments. The third indirect effect, of plant litter structuring soil microbial community composition, was minimized by the management of removing the plant biomass. Soil bacterial (16S rRNA) and soil fungal (18S rRNA) OTU abundances, plant species frequencies and soil factor chemical profiles were analyzed using multivariate statistics. Co-variation, or co-structures, between soil factor profiles and community compositions were determined using co-inertia analysis combining all four data sets. To our knowledge, this is the first study to apply co-inertia analysis to examine above- and below-ground community links in grasslands under long-term nutrient additions. Moreover, this is the first report on fungal community compositions sampled with 18S rRNA gene marker sequencing over these long-term fertilizer treatments.

## Results

Overall, the long-term fertilization treatments resulted in considerable shifts in the soil factor profiles and plant, bacterial and fungal community compositions, but generally not in community diversity or evenness indexes, with the exception of the plant community. We found different plant and soil fungal communities under the nitrogen-phosphate-potassium (NPK) and liming (L) treatments compared to the unfertilized control (C) treatment; furthermore, we found different soil bacterial communities and soil factor profiles under nitrogen (N) and L treatments compared to the unfertilized treatment. Interestingly, the plant-fungal phylum co-structure was significant, while that of the plant-bacterial phylum-level and fungal (phylum) and bacterial (phylum) comparisons were not. From pairwise co-inertia comparisons of communities, we identified taxonomic groups that co-varied in the N, NPK or L treatments.

### Treatment effects on plant and fungal community compositions and diversity

**Figure 1:**
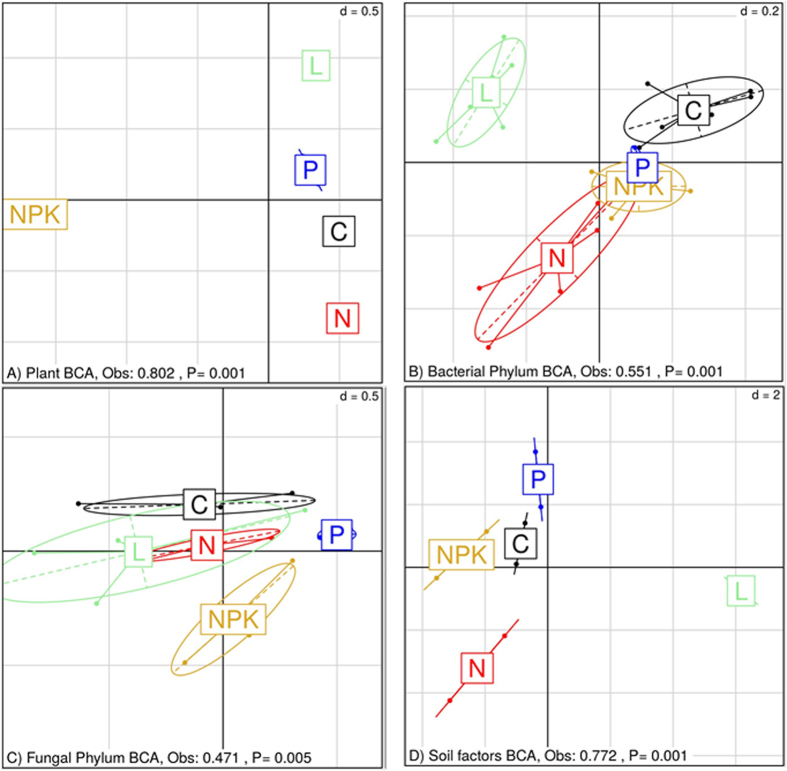
Between-Class Analysis (BCA) of the (**A**) plant, (**B**) bacterial, (**C**) fungal community compositions and (**D**) soil factor factors based on correspondence analysis (**A**–**C**) or principal components analysis (**D**) over the long-term control (**C**), liming (L), nitrogen (N), nitrogen-potassium-phosphorus (NPK) and phosphorus (P) treatments of the Ossekampen experiment are presented. Group significances were assessed by Monte-Carlo tests.

Between-Class Analysis (BCA) revealed similar structures in the plant and fungal communities across treatments. For these communities, the unfertilized control (C), nitrogen (N) and phosphorous (P) treatments clustered together while the liming (L) and N-P-potassium (NPK) treatments clustered separately (Fig. 1). From the BCAs of plant and fungal community compositions, 86% and 72% of total variation, respectively, could be attributed to treatment (Monte-Carlo test of groups, both P = 0.001).

Of the fungal samples across all treatments, 65 to 96% of the sequences could be classified at the phylum level. The five most abundant fungal groups represented an average of 96% of the proportion of classified sequences across the five treatments, and these groups were *Agaricomycotina* (average over all treatments 64%), *Saccharomyceta* (24%), “mitosporic” (3%), *Ascomycota* (3%) and *Glomerales* (2%). Fungal groups that differed significantly between the treatments were “mitosporic”, *Ascomycota, Glomerales, Paraglomerales, Chytridiales, Archaeosporales, Lobulomycetales* and *Rhizophydiales* (Tukey-Kramer, corrected p less than 0.05; Supplementary Table S1).

Fungal community evenness and diversity did not differ by treatment compared to the control treatment; similarly, plant community evenness did not differ by treatment compared to the control treatment (Supplementary Figs S1 and S2). However, the plant communities showed the lowest diversity in the NPK treatment while the highest diversity was present in the L treatment (Supplementary Fig. S2a). In addition to treatment and interaction with treatment, plant richness, bacterial diversity indices were significant effects on plant community compositions. For fungal community compositions, treatment and interaction with treatment as well as fungal diversity indices had significant effects (Supplementary Table S2).

### Treatment effects on bacterial community composition and diversity, and soil factor profiles

In contrast to the structures of plant and fungal communities across treatments that was revealed by BCA, the bacterial communities and the soil factors in the C, P and NPK treatments were grouped together while the L and N treatments each formed separate clusters (Fig. 1). The treatments could explain 55% and 72% of total variation in the BCAs of bacterial community compositions and soil factors, respectively (Monte-Carlo tests, P = 0.001). Of the 24 soil factors, 21 differed significantly between treatments; only the soil factors As, Cr and Na did not differ between treatments (Supplementary Fig. S3). When the soil factors were divided into subsets of micronutrients, macronutrients and structural factors BCA revealed that treatment could explain 71% (P = 0.001), 83% (P = 0.001) and 74% (P = 0.002) of the variation in each soil factor profile subset, respectively (Supplementary Fig. S4). Furthermore, while all treatments were distinct in the macronutrient profiles, only the L treatment was distinct when looking at micronutrient and structural factor profiles.

Between 79 and 91% of the bacterial sequences across all treatments could be classified at the Phylum level. Of the proportion of sequences classified within bacterial phyla, 97% were distributed across the treatments within the six most abundant Bacterial phyla, *Proteobacteria* (average across treatments 40%), *Acidobacteria* (22%), *Verrucomicrobia* (15%), *Actinobacteria* (13%), *Bacteroidetes* (3%) and *Planctomycetes* (1%). Significantly different bacterial phyla between treatments were *Proteobacteria, Acidobacteria, Verrucomicrobia, Actinobacteria, Bacteroidetes, Planctomycetes* and *Nitrospira* (Tukey-Kramer, corrected p less than 0.05, see Supplementary Table S3).

Bacterial community diversity and evenness was higher in the L treatment compared to the control treatment at all Renyi alpha levels, but were the same among the other treatments (Supplementary Figs S1 and S2). In addition to treatment and interaction with treatment, bacterial diversity (Shannon and Inverse Simpson indexes) and plant richness had significant effects on bacterial community compositions (Supplementary Table S3).

### Plant, bacterial and fungal community co-structures with soil factors

Co-inertia analysis allowed us to find the global similarity of the structures imposed by the long-term fertilization treatments on the grassland components. Co-structures resulting from the community-soil factor comparisons were each significant (Table 1). Further, we sub-categorized the soil factors into macronutrients (total K, total P, extractable K, P, NO_3_^-^, total N, NH_4_^+^, extractable N, C and OM), micronutrients (As, Cd, Cr, Ni, Al, Mg, Mn, Na, Pb and Zn) and structural components (pH, moisture), and conducted co-inertia analysis between communities and the soil factor subsets. Each of the community comparisons with soil factors subsets (Table 1).

### Plant, bacterial and fungal co-structures and co-variates

**Figure 2:**
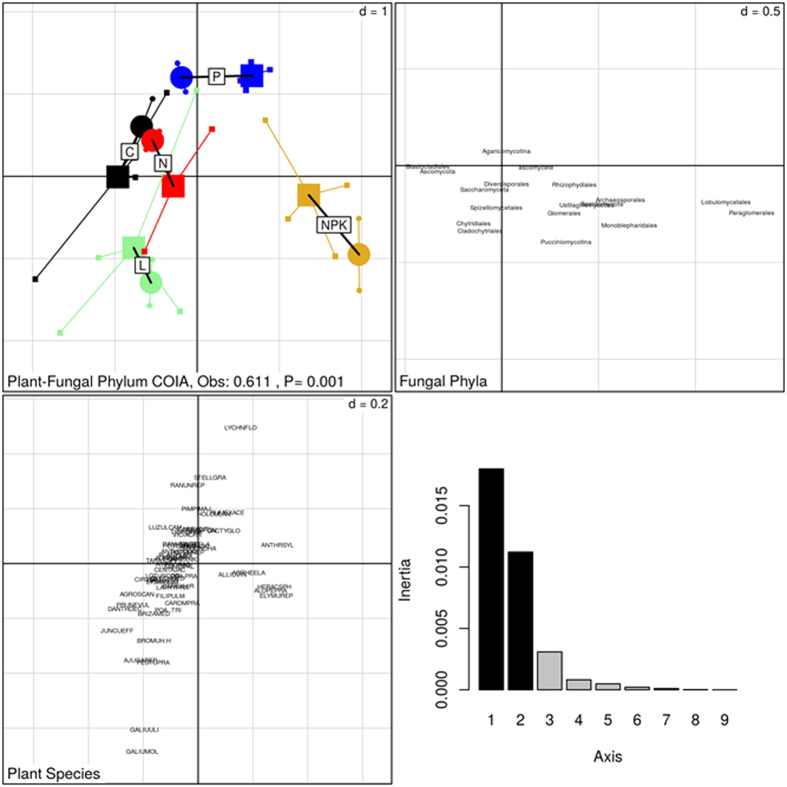
Co-inertia analysis (COIA) between correspondence analysis of plant and fungal community composition across the long-term control (C), liming (L), nitrogen (N), nitrogen-potassium-phosphorus (NPK) and phosphorus (P) treatments of the Ossekampen experiment (cumulative projected inertia = 86%). Significance of co-structure was assessed by a Monte-Carlo test. Circle = Plant; Square = Fungi.

**Figure 3:**
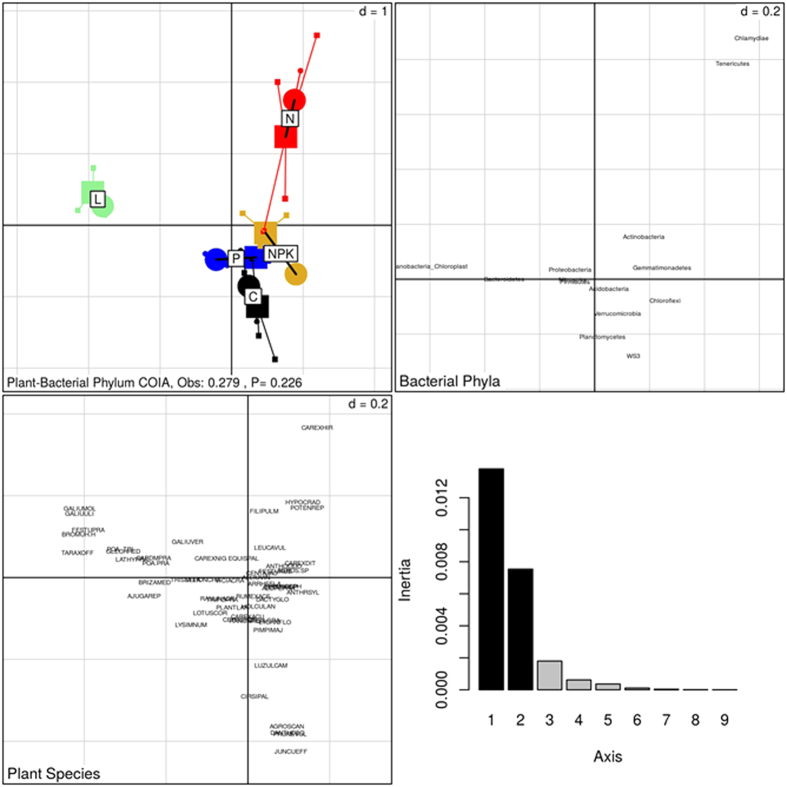
Co-inertia analysis (COIA) between correspondence analysis of plant and bacterial community composition across the long-term control (C), liming (L), nitrogen (N), nitrogen-potassium-phosphorus (NPK) and phosphorus (P) treatments of the Ossekampen experiment (cumulative projected inertia = 88%). Significance of the co-structure was assessed by a Monte-Carlo test. Circle = Plant; Square = Bacteria.

**Figure 4:**
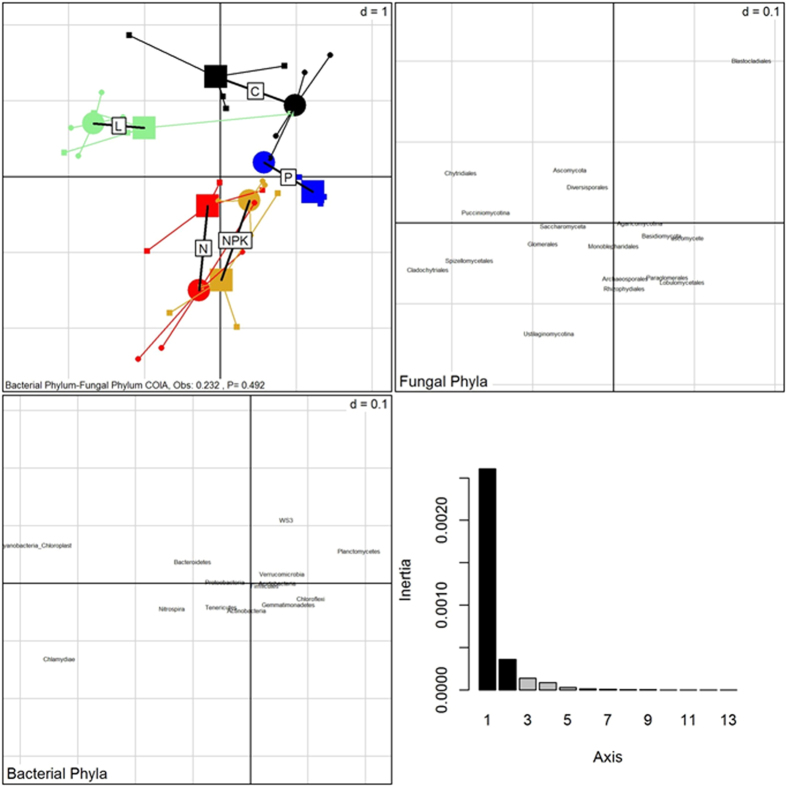
Co-inertia analysis (COIA) between correspondence analysis of bacterial and fungal community composition across the long-term control (C), liming (L), nitrogen (N), nitrogen-potassium-phosphorus (NPK) and phosphorus (P) treatments of the Ossekampen experiment (cumulative projected inertia = 92%). Significance of the co-structure was assessed by a Monte-Carlo test. Circle = Bacteria; Squere = Fungi.

Co-inertia analysis identified significant co-structures between the plant and fungal communities (Fig. 2) but not the plant and bacterial (Fig. 3) nor the bacterial and fungal (Fig. 4) communities. The first two co-inertia axes captured 86, 88 and 92% of the total variance in the plant-fungal, plant-bacterial and bacterial-fungal comparisons, respectively. The co-inertia analysis additionally identified taxonomic groups that most contributed to each co-inertia axis, i.e. groups that contributed the most to the total co-variance between samples (summarized in Table 2).

In the plant-fungal co-inertia (Fig. 2), the second co-inertial axis separated the NPK treatment from the other treatments, and this distinction was associated with Heracsph (herb), Anthrsyl (herb), Alopepra (dominant grass) and Elymurep (grass) plant species and *Monoblepharidales, Basidiomycetales, Ascomycetales* and *Paraglomerales* fungal groups. The P treatment was separated by the first co-inertia axis, and this was driven by Cerasfon (grass), Holculan (grass), Dactyglo (grass) and Luzulcam (sedge) plant species but not clearly any fungal groups. Last, Festupra (grass), Galiumol (herb), Bromuh H (grass), Glechhed (herb), Galiuuli (herb) and Ajugarep (herb) plant species and *Pucciniomycotina, Cladochytriales, Chytridiales* and *Spizellomycetales* fungal groups co-varied in the L treatment.

In the plant-bacterial co-inertia (Fig. 3), the NPK treatment was separated by the first co-inertia axis, and Anthrsyl (herb), Heracsph (herb), Alopepra (grass) and Elymurep (grass) plant species co-varied with *Nitrospira* bacterial phyla. The second co-inertia axis separated the N treatment from the other treatments, and this distinction was related to Carexhir (sedge), Carexdit (sedge), Juncuart (sedge) and Filipulm (herb) plant species and the *Tenericutes* bacterial phylum. Last, the L treatment grouping in the co-inertia between plant and bacterial ordinations was driven mainly by Bromuh H (grass), Galiumol (herb), Galiuuli (herb) and Glechhed (herb) plant species and WS3 and *Bacteroidetes* bacterial Phyla.

In the bacterial-fungal comparison (Fig. 4), treatments were not distinctly clustered. However, the L treatment was weakly separated by the first co-inertial axis, and the co-variation was driven by *Pucciniomycotina, Cladochytriales, Spizellomycetales* and *Chytridiales* fungal groups and the OD1 bacterial group. Furthermore, the N treatment was weakly separated by the second co-inertial axis, and this separation was driven by variation in the *Tenericutes* bacterial Phylum and the fungal groups *Ustilaginomycotina* and *Rhizophydiales*.

## Discussion

Here we explored indirect effects (pH and composition of other communities but not biomass of the plant community) and the direct effect of nutrient additions on the composition of three communities (plant, soil bacterial and soil fungal) of an improved grassland. Changes in the relative abundances of many taxonomic groups within one community resulted in community-level composition changes in some treatments. Our first hypothesis was supported, in that the bacterial communities were altered in the nitrogen (N) treatment but not phosphorus (P) nor NP-potassium(K) treatments, and the plant and fungal communities were altered in the NPK but not the P nor N treatments, compared to the unfertilized treatment. Combining the treatment groupings of each grassland community and the links between each community and macronutrients, we confirmed the direct effect of the nutrient additions on the communities.

The nutrient addition and mowing management practice provided selective pressures that drove changes in the relative abundances of some plant, bacterial and fungal groups. The treatments represented new habitats that allowed some functional types to succeed; for instance, in the NPK treatment, faster-growing individual plants better able to compete for light and uptake nutrients were most successful^14^. This trait selection led to changes in the relative abundances of grasses in the NPK treatment. We found a direct effect of the long-term N fertilizer treatment on the bacterial communities, compared to the unfertilized treatment, while no direct effect was found in the NPK treatment despite an equivalent rate of N application. In our previous work and here, we found so-called copiotrophic bacterial groups that were functionally adapted to the N additions to increase in relative abundance in the N treatment, which has been found at other grassland sites under long-term N additions^15^,^16^. Furthermore, the NPK treatment, but not the N treatment, had a direct effect on plant and fungal community compositions compared to the unfertilized treatment.

Community compositions can be altered indirectly by nutrient additions through the effect on pH. The liming (L) treatment changed the soil pH, which alters other soil factors, such as Al concentration and micronutrient availability^17^. Thus any changes in L community composition were due to the increase in pH and mowing management practice. Our second hypothesis, that each community would be affected by the L treatment when compared to the unfertilized control treatment, and that each community would show a link to soil factors, was supported. Each community was altered in the L treatment compared to the unfertilized treatment, supporting pH changes and related edaphic factor changes as a major driver of bacterial and fungal composition changes^18^,^19^,^20^. As shown previously, the near-neutral pH of the L treatment soils increased nutrient availability resulting in native plant species recolonizing the L plots and overall increased plant richness^14^. The L treatment was characterized by high plant diversity and evenness and additionally by non-pathogenic nematode and earthworm biomass, which characterize fertile (e.g. productive) grasslands^21^.

Here we considered that some of the changes in the relative abundances of taxonomic groups reflected not only functional adaptation to the treatment but a link with taxonomic groups from other communities. Therefore, we explored the indirect effect of nutrient additions on the composition of a community through the composition of another community. In other words, these were community links apart from the direct effects of the nutrient additions. Most interestingly, our third hypothesis, that each community would be significantly linked to each other, was not supported. Community-level composition links were not found for plant and bacterial, and fungal and bacterial communities. Thus, the indirect effect of nutrient additions altering community compositions through changes in other community compositions was supported between plant and fungal (phylum) communities, and between each community when the soil microbial communities were compared at a lower taxonomic level. Recent work has found that the beta diversity, or site differences, of grassland soil microbial communities are predicted by the beta diversity of the overlying plant communities^12^. The authors also found that the beta diversities of plants and soil microbes were driven mutually by mean annual temperature and the C:N ratio. We speculate that our NPK bacterial communities may have shifted in association with the plant community if the plant biomass had remained on the fields. In support, Millard and Singh^22^ suggested that while plant community composition drives fungal community composition, the bacterial community structure is more influenced by SOM quality, hence driven indirectly by plant community composition via plant biomass.

The acquisition-conservation trade-off hypothesis states that under soil resource limitation, the plant community is more dependent on the below-ground community for nutrients, resulting in more ecological links between plant and microbial species^23^. Conversely, during high nutrient availability, fewer links should be found between the plant and soil microbial communities, with plants less dependent on nutrients from fungal or bacterial sources. Here, plant community diversity decreased in the NPK treatment compared to the unfertilized treatment and grasses were better adapted to the high nutrient conditions. Consequently, fungal groups associated with the grasses might have shifted under the high nutrient conditions, thereby changing fungal community composition in the NPK treatment. Selection of the grasses on fungal groups that increase grass root nutrient uptake may have occurred, although whether the plant-fungal link describes positive or negative interactions is beyond the scope of the current study.

One interesting NPK fungal co-variate was *Paraglomerales*, which are a fungal group that includes arbuscular mycorrhizal fungi (AMF); these fungi form hyphal networks in association with plant roots to aid plant absorption of P. There is evidence that under conditions of high P availability, plants form fewer AMF associations because available P is directly absorbed from soil^24^,^25^, and this was supported in our results as the P treatment fungal communities had the lowest proportions of AMF compared to the other treatments. However, in the NPK treatment with N and K added, there was a high soil Al-content, which may block P uptake by plant by fixing it in the soil^26^. Thus, the NPK plants may be investing in AMF associations in order to improve soil P uptake despite the high Al content. Alternately, plant-AMF interactions can become competitive under high-nutrient conditions, and these results may indicate a negative interaction^27^,^28^.

An interesting bacterial NPK co-variate was the *Nitrospira* bacterial phyla, which are a group of autotrophic, nitrite-oxidizing bacteria^29^. This group was abundant in the NPK treatment but not in the N treatment, compared to the control treatment. *Nitrospira* are classified as K-strategists, that is, slow-growing, oligotrophic and with low affinity for N substrates^30^. Similarly, *Nitrospira* relative abundances decreased in grasslands fertilized for 27 years and agricultural fields under 8 years of N fertilization consistent with the rate in our plots^15^. However, under high nutrient fertilization, plant competition for macro-nutrients with bacteria may keep the soil nutrient status poor; thus, we hypothesize that the *Nitrospira* co-varied with NPK plants due to the relatively low-nutrient conditions available in these soils for the bacterial community, in contrast to the high N conditions available in the N treatment.

In the N treatment comparisons, microbial co-variates included potential pathogenic taxa. For instance, the N treatment co-variate *Rhizophydiales* are a zoosporic fungal taxa that are found in soils as pathogens and decomposers^31^. The *Tenericutes* bacterial Phyla encompasses the phytoplasmas, which are regarded as plant pathogens, infecting up to 98 plant families^32^. The *Ustilaginomycotina* are a group of plant parasitic fungi, also known as smut fungi^33^. These results suggest that plant health may be negatively impacted under N fertilization, with consequences on the long-term stability of plant communities regularly fertilized with N.

The current study presents a way to investigate concurrently the plant, bacterial and fungal communities using a not-widely used statistical analysis. However, we acknowledge that our results are limited by the low statistical power induced by having only two plots per treatment for the plant and soil factor data. We sampled soil samples from independent sampling areas within each of the two plots, resulting in two samples within each plot for the fungal data (n = 20), and three samples within each plot for the bacterial data (n = 27).

In summary, we explored direct (long-term fertilizer treatments) and indirect (pH and composition of another community) effects of nutrient additions on plant, soil microbial community compositions from grassland. This is, to our knowledge, the first study of the topsoil fungal community composition to long-term inorganic fertilization treatments in grasslands using the 18S rRNA gene marker. In addition, this is the first study to examine concurrently the above- and below-ground community compositions in an improved grassland using co-inertia analysis. Nitrogen treatment had a direct effect on bacterial community compositions and soil factors while NPK treatment had a direct effect on plant and fungal community compositions. Co-inertia results highlighted the link between plant and fungal community compositions, suggesting that indirect effect of nutrient additions on plant community compositions are observed due to fungal community compositions, or vice versa, while the same is not true for plant and bacterial communities, nor for fungal and bacterial communities. However there was also an effect between plant and bacterial community diversities impacting the plant and bacterial compositions, respectively, suggesting that these communities also are linked. To examine the indirect effects of nutrient additions on the grassland communities, we necessarily used symmetric co-inertia analyses; therefore, it should be emphasized that these results imply correlations and not causation. In addition to community-level links, we found potential association between plant, bacterial and fungal taxonomic groups in the N, NPK and L treatments that can be explored in future studies.

## Material and Methods

### Site description

The Ossekampen Grassland Experiment fields were established in 1958 in species-rich meadows on heavy-clay soil in Wageningen, The Netherlands with coordinates 51 degrees 58′15″N; 5 degrees 38′18″E. Prior to the experiment, the land was grazed and had been used in alternate years for haymaking. Fertilizer treatments of chalk (L; 1000 kg CaO ha^−1^ yr^−1^), nitrogen (N; ammonium nitrate, 100 kg N ha^−1^ yr^−1^), phosphate (P; superphosphate, 22 kg P ha^−1^ yr^−1^) and NP-potassium (NPK; ammonium nitrate, superphosphate and potassium sulfate, 160 kg N ha^−1^ yr^−1^, 33 kg P ha^−1^ yr^−1^ and 311 kg K ha^−1^ yr^−1^) were applied to duplicate 40 m^2^ (16 m × 2.5 m) plots annually since 1958 34. The control treatment fields were established without nutrient amendment. The five treatment fields were mown twice a year, in July and in October, and the biomass was removed toward the 2.5 m ends of the plots to prevent seeds from draining into the other treatment plots. Treatment and control plots were separated by unfertilized 2.5 m buffer strips, which were similarly maintained by mowing.

### Plant sampling regime

The botanical composition was measured in September 2011, after peak plant growth^14^. Briefly, fifty samples of 25 cm^2^ were clipped from each plot (2 plots × 5 treatments = 10 plant samples). The presence of each species was recorded to determine its frequency percentage (i.e. the proportion of 50 samples in which the species was present). In addition, the first, second and third most abundant species were recorded in each sample by visual estimation. The Dry Weight Rank (DWR) method was developed to estimate the species composition of grassland swards on a dry weight basis^35^. The DWR method calculates for each species its dry weight proportion (DW percentage for species A) from the percentages of cases the species takes the first (A1%), second (A2%) and third (A3%) rank. These proportions are multiplied by 0.702, 0.211 and 0.87, respectively, culminating in the following equation: DWA% = 0.702 (A1%) + 0.211 (A2%) + 0.087 (A3%). This method was tested with different sampling methods, including small samples, and it was concluded that the DWR method is well suited for studying vegetation changes in old, floristically diverse grasslands^36^, such as ours. For each plant species, the functional classification was noted (grass, herb, legume or forb).

### Soil sampling and total DNA extraction

In September 2011, nine random soil core samples (10 cm depth, two cm diameter) were taken from three independent areas within each duplicate plot of the five treatment fields. The nine soil cores were homogenized per area, sieved through 5 mm pores stored at −80 °C for molecular analyses (see below) or stored at −20 °C for one night before being sent for physico-chemical analysis as follows. The soil samples were combined per plot for physicochemical analyses for a total of 10 soil factor samples (3 areas homogenized to 1 sample × 2 plots × 5 treatment fields). These samples were dried at 60 °C to measure moisture content. Soil pH, extractable N, total carbon and nitrogen concentrations, available potassium, phosphate and sulfur, total organic matter and available trace elements such as Al, As, Cd, Cr, Cu, Fe, Mg, Zn, Mn, Na, Ni and Pb were analyzed at the Soil Science Department of Wageningen University (Supplementary Table S4)^37^. For the molecular investigations, total DNA was extracted from 0.3 g fractions of each soil sample (3 areas × 2 plots × 5 treatments = total 30 soil samples). The Power Soil kit (MolBio, Carlsbad, CA) protocol was followed with the modification of 5.5 m s^−1^ for 10 min. bead beating. Total DNA concentrations were measured using an ND-1000 spectrophotometer (Nanodrop, Wilmington, DE).

### 16S rRNA amplicon library preparation

The V4 region of the 16S rRNA gene marker was amplified from each sample, for a total of 30 bacterial samples (3 areas × 2 plots × 5 treatments). Amplicons for barcoded pyrosequencing were obtained using the forward primer 515F (5′-GTGCCAGCMGCCGCGGTAA-3′) and the reverse primer 806R (5′-GGACTACVSGGGTATCTAAT-3′). The 515F primer included the Roche 454-A adapter, a 10-bp barcode and a GT linker, and the reverse primer included the Roche 454-B adapter, the same 10-bp barcode as the 515F primer, and a GG linker. Amplification reactions were performed using 5 micromolar of each forward and reverse primer, 5 mM dNTPs (Invitrogen, Carlsbad, CA), 1 unit of *Taq* polymerase (Roche, Indianapolis, IN), and 1 microliter of sample DNA as the template in a total volume of 25 microliters. The PCR was conducted with an initial incubation of 5 min. at 94 °C, followed by 25 cycles of 1 min. at 94 °C, 1 min. at the annealing temperature of 53 °C and 1 min. at 72 °C, followed by a final incubation of 10 min. at 72 °C. Each sample was amplified in four reactions, and resulting amplification products were pooled to achieve equal mass concentrations in the final mixture.

### 18S rRNA amplicon library preparation

The 18S rRNA gene marker was amplified from two of the three soil samples per plot, for a total of 20 fungal samples (2 areas × 2 plots × 5 treatments). The 18S rRNA gene marker was amplified from the total community DNA samples using the primers FR1 (5′-AICCATTCAATCGGTAIT-3′) and FF390.1 (5′-CGWTAACGAACGAGACCT-3′) based on published methods^38^. The Roche MID tag IDs 24 to 26 and 61 to 69 were added to barcode the samples. Amplification reactions were performed using 5 micromolar of each primer, 2 mM dNTPS (Invitrogen, Carlsbad, CA), 0.5 microliters of BSA, 10 PCR buffer, 0.56 units of Fast Start Exp-Polymerase and 1 microliter of sample DNA template in a total reaction volume of 25 microliters. The PCR was conducted with initial incubation of 5 min at 95 °C C followed by 25 cycles of 30 sec. at 95 °C, 1 min. at the annealing temperature of 57 °C, 1 min. at the extension temperature of 72 °C, followed by a final extension for 10 min. at 72 °C. Each sample was amplified in two reactions, and resulting amplification products were pooled to achieve equal mass concentrations in the final mixture.

### 16S and 18S rRNA amplicon library sequencing and processing

Amplification products were cleaned using the QIAquick PCR Purification Kit following the manufacturer’s instructions (Qiagen, Valencia, CA). The purified 16S and 18S rRNA amplicon products were sequenced on the Roche 454 FLX Titanium platform (Macrogen Inc, South Korea). The 18S rRNA sequence data was processed using QIIME v1.3.0-dev on a local installation of Galaxy^39^. The 16S rRNA sequence data was processed using MOTHUR on a local 64-node server running Ubuntu (Ubuntu-14.04-trusty). Low-quality sequences that were less than 150 bp in length or that had an average quality score of less than 25 were removed. Denoising and chimera checking were accomplished using USEARCH and UCHIME^40^,^41^. Operational Taxonomic Units (OTUs) were identified using USEARCH with a phylotype defined at 97% sequence similarity level. For the 18S rRNA dataset, representative phylotype sequences were taxonomically assigned against the SILVA 104 database^42^ using BLAST at an e-value of 0.001. For the 16S rRNA dataset, representative phylotype sequences were assigned to the SILVA database using the RDP classifier v10.

### Statistical analyses

Statistical analyses were conducted in R v3.1.1 (Team, R. C. R: A language and environment for statistical computing). The sequenced datasets (16S and 18S rRNA) were handled in R with the “phyloseq” package (McMurdie, P. & Holmes, S. phyloseq: an R package for reproducible interactive analysis and graphics of microbiome census data). Further methods can be found in the Supplementary Methods.

#### Treatment effects on each component

To determine the effect of treatment on the plant (n = 10), bacterial (n = 27) and fungal (n = 20) community compositions and the soil factor profiles (n = 10), the “ade4” R package was used^43^. Between-class analysis (BCA) was applied to explore the dissimilarity between treatments within each community (plant, bacterial, fungal) or soil factors (environmental). Correspondence Analysis (CA) was applied to each community dataset while correlation-based Principal Component Analysis (PCA) was used for the soil factors^44^. A Monte-Carlo test of the treatment groups was conducted using 999 random permutations of sample rows. Ordination was used to visualize the BCA results. To determine the effect of treatment on the soil factors or community diversities (for calculations, see Supplementary Methods), multiple group comparisons were conducted in R. Of the 27 measured soil factors, the variable Cu was removed for having the same value for all treatments; similarly, the variables PO_4_ and Fe were removed for containing values that fell below the detection limit for four or more treatments (Table S4). The remaining 24 soil factors were standardized and checked for normality with the Shapiro-Wilk test (normal variables: total N, Al, Mg, Mn, Na, Pb, Zn, NH_4_, extractable N, C.1, OM, C.2; non-normal variables: total K, total P, As, Cd, Cr, K, Ni, P, S, NO_3_, pH, moisture). The Kruskal-Wallis H or ANOVA statistical test was applied to compare the non-normal and normal variable median and means, respectively, between treatments. Post-hoc comparisons (two-tailed) were conducted using Tukey’s HSD test (alpha level = 0.05) or Dunn’s method (alpha level = 0.10), respectively, and boxplots were constructed.

#### Between-component analyses

To examine the common structure in plant, bacterial and fungal community compositions and soil factor profiles across the long-term treatments, co-inertia analysis was conducted. The “RV.test” R function was used to perform a Monte-Carlo test on the sum of eigenvalues in the co-inertia^45^. For the co-inertia analyses, the community and soil factor datasets were required to have the same number of samples per treatment. Because we had two plant samples and two soil factor samples per treatment, for these datasets we duplicated the values to result in four plant samples and four soil factor samples per treatment. First, co-inertia was performed for paired combinations of community CA ordinations and the soil factor PCA ordination, and visualizations were constructed. Additional co-inertia analyses were carried out between the community CA ordinations and PCA ordinations of three subsets of the soil factor dataset: macro-nutrients (total K, total P, extractable K, extractable P, NO_3_^−^, Nt, NH_4_^+^, extractable N, C and OM), micro-nutrients (As, Cd, Cr, Ni, Al, Mg, Mn, Na, Pb and Zn) and structural components (pH, moisture); the community-soil factor subset co-inertia analyses were visualized. To determine whether diversity of another community as well as Treatment had an effect on the composition of the original community, we tested these hypotheses using PERMANOVA (“vegan” R package).

## Additional Information

**Accession codes:** European Nucleotide Archive study accession number PRJEB11582.

**How to cite this article**: Cassman, N. A. *et al*. Plant and soil fungal but not soil bacterial communities are linked in long-term fertilized grassland. *Sci. Rep.* **6**, 23680; doi: 10.1038/srep23680 (2016).

## Supplementary Material

Supplementary Information

## Figures and Tables

**Table 1:** 

Co-inertia comparison		Cumulative projected inertia (%)	Observed Rv	P
Plant species	All soil factors	85	0.763	0.001
	Micronutrients	90	0.646	0.001
	Macronutrients	85	0.687	0.001
	Structural	100	0.650	0.001
Bacterial phyla	All soil factors	97	0.346	0.004
	Micronutrients	97	0.251	0.029
	Macronutrients	96	0.400	0.001
	Structural	100	0.221	0.087
Fungal phyla	All soil factors	87	0.437	0.001
	Micronutrients	89	0.300	0.046
	Macronutrients	93	0.496	0.001
	Structural	100	0.308	0.025

Co-inertia analysis results from community-soil factors and soil factor subset comparisons from the Ossekampen experiment (n = 20 per dataset). Significance of co-structures was assessed by Monte-Carlo tests.

**Table 2:** 

	Co-variates within treatment			
Community	N	NPK	P	L
Plant	Filipulm (h)	Heracsph (h)	Cerasfon (g)	Galiuuli (h)
	Carexhir (s)	Anthrsyl (h)	Holculan (g)	Glechhed (h)
	Carexdit (s)	Alopepra (g)	Dactyglo (g)	Ajugarep (h)
	Juncuart (s)	Elymurep (g)	Luzulcam (s)	Galiumol (h)
				Bromuh H (g)
				Festupra (g)
				Poa..tri (g)
Bacteria	Tenericutes	Nitrospira	–	Bacterioidetes
				WS3
				OD1
Fungus	Ustilaginomycotina	Paraglomerales	–	Pucciniomycotina
	Rhizophydiales	Basidiomycetales		Cladochytriales
		Ascomycetales		Chytridiales
		Monoblepharidales		Spizellomycetales

List of co-variate taxonomic groups from pairwise co-inertia analysis of plant, bacterial and fungal community composition from the Ossekampen experiment. Taxonomic groups that contributed to the co-inertia axes clearly separating the nitrogen (N), N-phosphate-potassium (NPK), P and chalk (L) treatments are listed. Plant functional group information is included (s = sedge, h = herb, g = grass).
