# Supplementary material for: Plant and soil fungal but not soil bacterial communities are linked in long-term fertilized grassland
Source: Sci Rep. 2016 Mar 29;6:23680. doi: 10.1038/srep23680 (PMC4810522; doi:10.1038/srep23680)
Supplement: Supplementary Information [file srep23680-s1.pdf]

Plant and soil fungal but not soil bacterial communities are linked in long-term fertilized grassland

Noriko A Cassman[1,2], Marcio F A Leite[1,3], Yao Pan[1], Mattias de Hollander[1], Johannes A van Veen[1,2], and Eiko E Kuramae[1,*]

[1]Netherlands Institute of Ecology NIOO-KNAW, Department of Microbial Ecology, Wageningen, 6708 PB, Netherlands

[2]Leiden University, Department of Biology, Leiden, 2311 EZ, Netherlands

[3]Maranhão State University, Department of Agroecology, São Luís Maranhão, 65055-000, Brazil

[*]E.Kuramae@nioo.knaw.nl

Supplementary Table S1. Fungal community compositions in the long-term unfertilized control (C), liming (L), nitrogen (N), N-phosphate-potassium (NPK) and P treatments of the Ossekampen experiment. Fungal phyla were included if the average proportion of classified sequences was above 1% in at least one treatment.

Fungal Phyla	Effect size	Average proportion of classified sequences in treatments (%)									
		C		L		N		NPK		P	
Agaricomycotina	0.506	61±17		46±21		62±10		63±9		89±3	
Saccharomyceta	0.405	28±15		32±16		30±10		22±7		7±2	
**“mitosporic”+**	0.679	2±2	a*	8±3	b	1±0	a	5±2	ab	1±0	a
**Ascomycota**	0.740	6±2	a	5±0	a	3±1	ab	1±1	b	1±1	b
**Glomerales**	0.682	1±0	a	3±2	b	2±1	ab	4±1	b	1±0	a
**Paraglomerales**	0.768		a		a		a	1±1	b		a
**Chrytidiales**	0.608		a	2±1	b		a		a		a
Pucciniomycotina	0.208			1±2				1±1			

+bolded phyla indicate significant difference among treatments (ANOVA, corrected $p<0.05$ from STAMP analysis)
*Similar letters represent no significant differences between treatments (Tukey-Kramer, 95% CI, Benjamini-Hochberg FDR multiple test correction)

Supplementary Table S2. PERMANOVA test results of treatment and diversity effects on plant, bacterial and fungal community compositions in the Ossekampen experiment.

Community	Source of Variation	Pseudo-F	p-value
Plant species	**Treatment**	**3.1444**	**0.045**
	**Plant Richness**	**14.9652**	**0.001**
	**Interaction**	**3.4186**	**0.043**
	**Treatment**	**60.824**	**0.001**
	**Bacterial Shannon**	**3.975**	**0.014**
	**Interaction**	**3.371**	**0.002**
	**Treatment**	**51.359**	**0.001**
	**Bacterial Inverse Simpson**	**2.901**	**0.053**
	**Interaction**	**2.571**	**0.016**
	**Treatment**	**42.879**	**0.001**
	Fungal Shannon	1.048	0.351
	**Interaction**	**2.077**	**0.041**
	**Treatment**	**35.710**	**0.001**
	Fungal Inverse Simpson	0.862	0.450
	Interaction	1.314	0.245
Bacterial phyla	**Treatment**	**33.772**	**0.001**
	**Plant Richness**	**15.379**	**0.001**
	**Interaction**	**6.753**	**0.001**
	**Treatment**	**20.4142**	**0.001**
	**Bacterial Shannon**	**8.8261**	**0.001**
	**Interaction**	**3.2105**	**0.009**
	**Treatment**	**13.0175**	**0.001**
	**Bacterial Inverse Simpson**	**6.1752**	**0.006**
	Interaction	1.0046	0.482
	**Treatment**	**9.1368**	**0.001**
	Fungal Shannon	0.9903	0.395
	Interaction	0.7958	0.643
	**Treatment**	**8.5066**	**0.001**
	Fungal Inverse Simpson	0.7469	0.473
	Interaction	0.6123	0.784
Fungal phyla	**Treatment**	**5.3954**	**0.007**
	Plant Richness	0.6262	0.458
	**Interaction**	**3.1178**	**0.048**
	**Treatment**	**3.3005**	**0.033**
	Bacterial Shannon	0.6617	0.464
	Interaction	0.8668	0.548
	**Treatment**	**3.2082**	**0.057**
	Bacterial Inverse Simpson	0.4438	0.583
	Interaction	0.8226	0.582
	**Treatment**	**9.3895**	**0.002**
	**Fungal Shannon**	3.6953	0.067
	**Interaction**	**6.6252**	**0.005**
	**Treatment**	**9.0983**	**0.001**
	**Fungal Inverse Simpson**	**5.4546**	**0.023**
	**Interaction**	**5.8736**	**0.004**

Supplementary Table S3. Bacterial community compositions in the long-term unfertilized control (C), liming (L), nitrogen (N), N-phosphate-potassium (NPK) and P treatments of the Ossekampen experiment. Phyla were included if the average proportion of classified sequences was above 1% in one or more treatments.

Bacterial Phyla	Effect size	Average proportion of classified sequences (%)									
		C		L		N		NPK		P	
**Proteobacteria+**	0.836	35±2	a*	51±1	c	41±4	b	38±3	ab	37±2	ab
**Acidobacteria**	0.501	28±4	a	19±4	b	20±3	b	23±2	ab	23±2	ab
**Verrucomicrobia**	0.642	24±7	ac	8±2	b	13±4	bc	19±4	a	20±2	a
**Actinobacteria**	0.675	5±2	a	9±3	ab	18±5	c	12±2	b	11±1	ab
**Bacteroidetes**	0.900	3±0	a	8±1	b	2±1	a	2±1	a	3±0	a
Firmicutes	0.285	2±0		3±1		2±1		2±1		3±0	
**Planctomycetes**	0.709	2±0	abc	1±0	ab		ac	1±0	abc	1±0	ab
**Gemmatimonadetes**	0.527	1±0				1±0		1±1		1±0	

+bolded phyla indicate significant difference among treatments (ANOVA, corrected p<0.05 from STAMP analysis)

*Similar letters represent no significant differences between treatments (Tukey-Kramer, 95% CI, Benjamini-Hochberg FDR multiple test correction)

Supplementary Table S4. Additional information regarding the soil factors measured in the Ossekampen experiment. Horizontal lines group measurements from the same method.

Soil Factor	Soil Factor ID	Units	Method	Notes
Total K	Kt	mg/kg	F-AES	Aqua Regia digestion
Total N	Nt	g/kg	SFA-Nt/Pt	H_2SO_4-H_2O_2-Se digestion
Total P	Pt	mg/kg		
Al	Al	mg/kg	ICP-AES Thermo	0,01M $CaCl_2$ extraction
As	As	mg/kg	ICP-AES Thermo	
Cd	Cd	mg/kg	ICP-AES Thermo	
Cr	Cr	mg/kg	ICP-AES Thermo	
Cu	Cu	mg/kg	ICP-AES Thermo	Factor removed from dataset; values all the same
Fe	Fe	mg/kg	ICP-AES Thermo	Factor removed from dataset; values fell below detection limit
Extractable K	K	mg/kg	ICP-AES Thermo	
Mg	Mg	mg/kg	ICP-AES Thermo	
Mn	Mn	mg/kg	ICP-AES Thermo	
Na	Na	mg/kg	ICP-AES Thermo	
Ni	Ni	mg/kg	ICP-AES Thermo	
Extractable P	P	mg/kg	ICP-AES Thermo	
Pb	Pb	mg/kg	ICP-AES Thermo	
S	S	mg/kg	ICP-AES Thermo	
Zn	Zn	mg/kg	ICP-AES Thermo	
NH_4^+	NH_4^+	mg/kg	SFA-$CaCl_2$	
NO_3^-	NO_3^-	mg/kg	SFA-$CaCl_2$	
Extractable N	Nts	mg/kg	SFA-$CaCl_2$	
PO_4	--	mg/kg	SFA-$CaCl_2$	Factor removed from dataset; values fell below detection limit
C	C.1	mg/kg	SFA-TOC	
Organic matter	OM	%	baking oven	Loss on ignition (105-550°C)
C	C.2	g/kg	spectrophotometer	Kurmies
pH	pH	at 20±1˚C	pH-meter	pH-H_2O
Moisture	Moisture	% dry-matter	dry matter	moisture determination

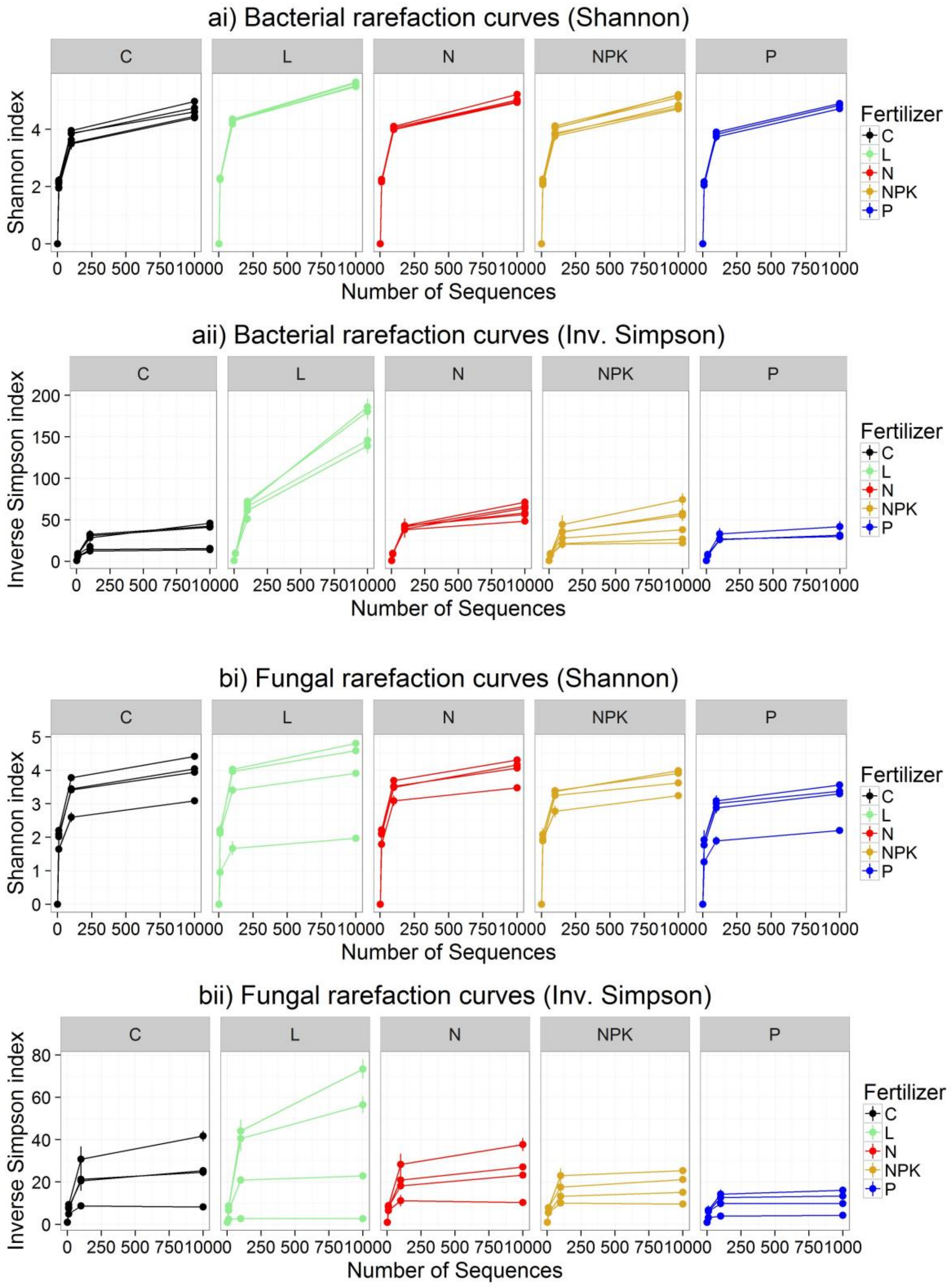

Supplementary Figure S1. Rarefaction curves of the sequenced communities from the Ossekampen experiment, including the a) bacterial and b) fungal communities, are presented using (i) Shannon and (ii) Inverse Simpson diversity indices. Legend: control (C), liming (L), nitrogen (N), nitrogen-potassium-phosphorus (NPK and phosphorus (P) fertilizer treatments.

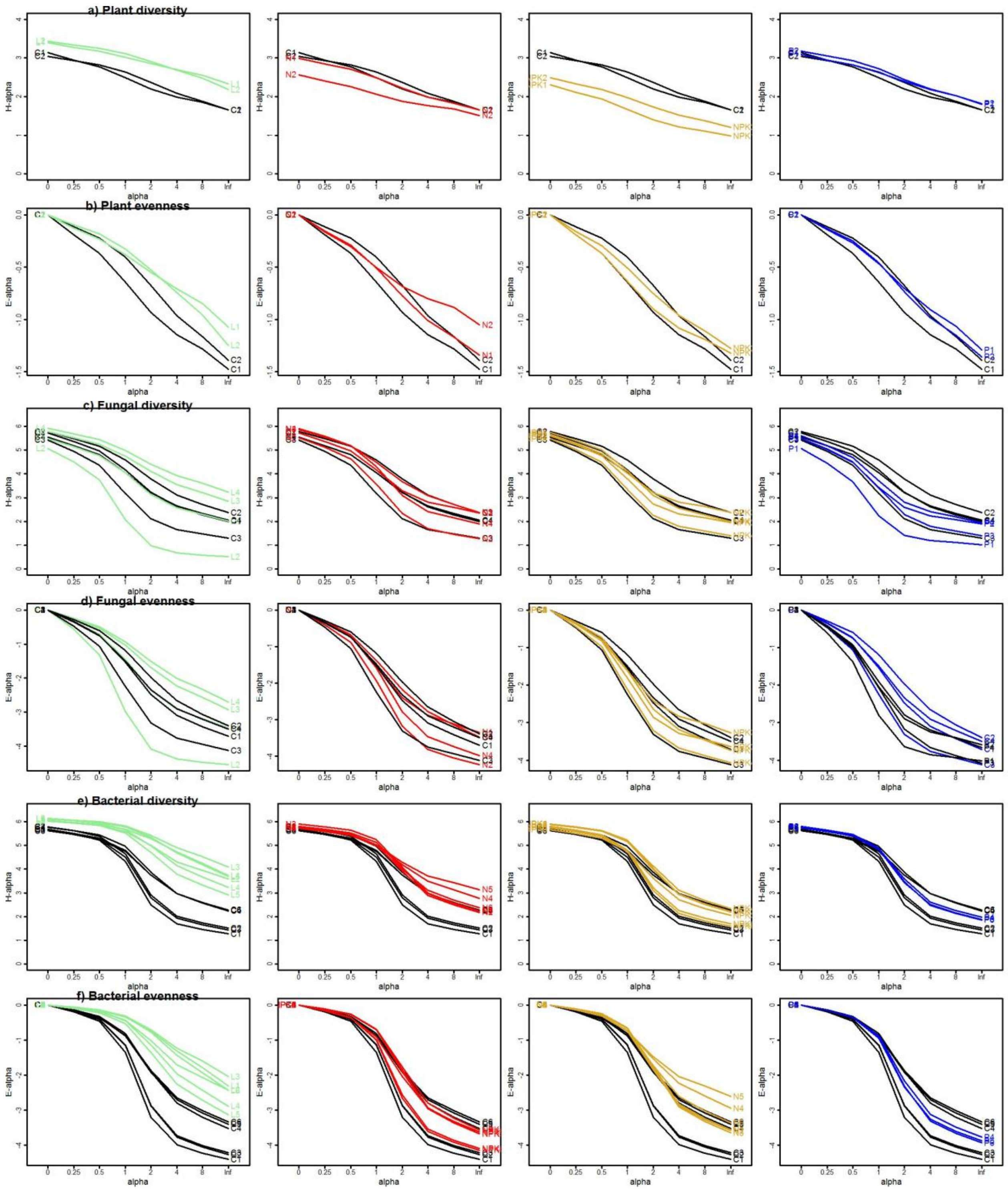

Supplementary Figure S2. Renyi diversities and evenness of a-b) plant (n=10), c-d) fungal (n=20) and e-f) bacterial (n=27) communities in the Ossekampen experiment. Legend: control (C), liming (L), nitrogen (N), nitrogen-potassium-phosphorus (NPK) and phosphorus (P) fertilizer treatments.

a) Non-normal soil factors and diversity indices

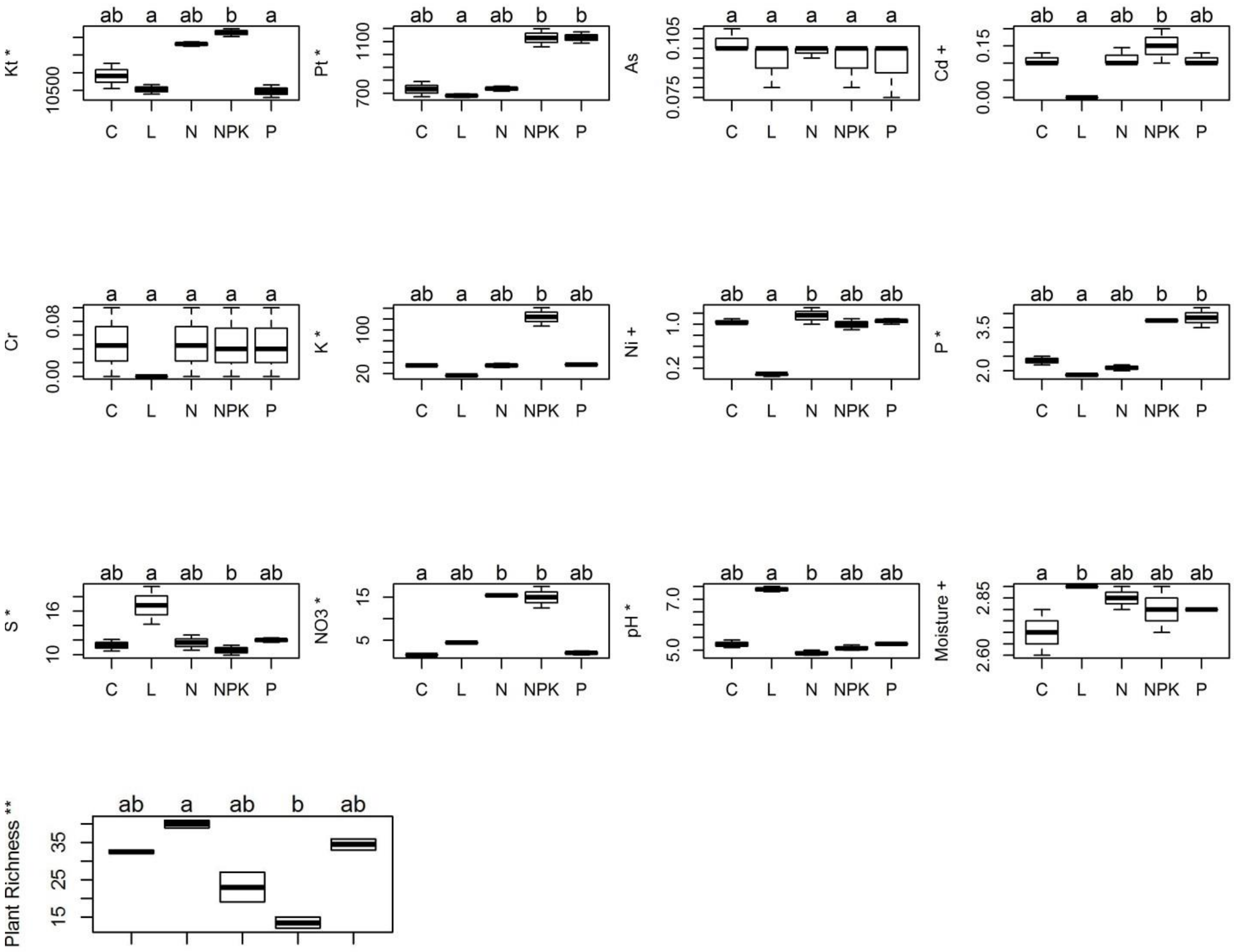

Kruskal-Wallis H test significance: ** p<0.01, * p < 0.05, + p < 0.1

Dunn’s post-hoc test significance: similar letters mean no difference at alpha level 0.10

b) Normal soil factors and diversity indices

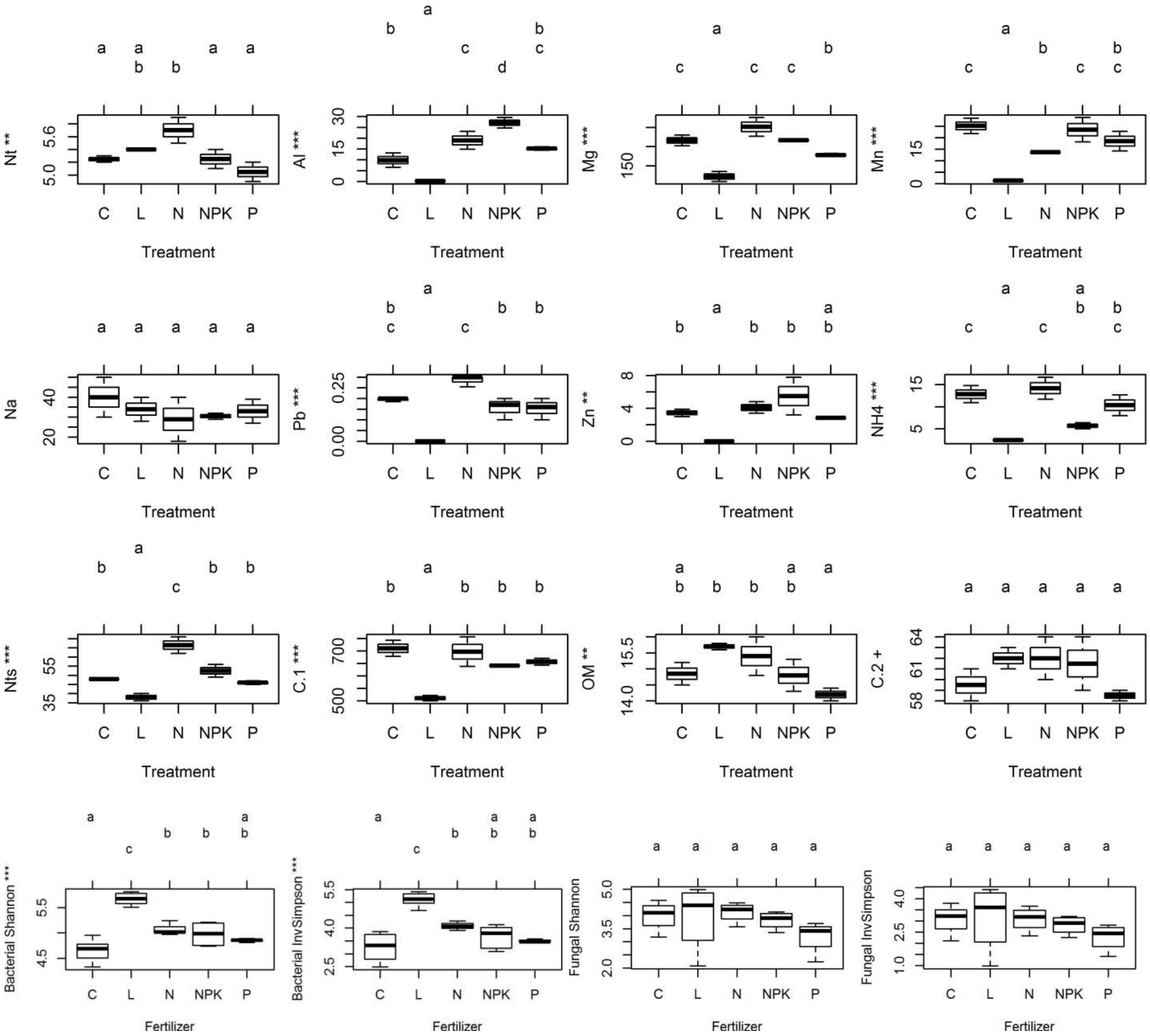

ANOVA test significance: *** $p < 0.001$, ** $p = 0.001$, * $p = 0.01$, + $p = 0.1$
Tukey-Kramer post-hoc test significance: similar letters represent no difference at alpha < 0.05

Supplementary Figure S3. Boxplots of the a) non-normal and b) normal soil physicochemical parameters and community diversity indices in the Ossekampen experiment. Legend: control (C), liming (L), nitrogen (N), nitrogen-potassium-phosphorus (NPK) and phosphorus (P) fertilizer treatments. Asterisks by variable names indicate significantly different mean or median values across all treatments from Kruskal-Wallis or ANOVA tests, respectively. Soil factor identifiers (IDs) are listed in Table S4.

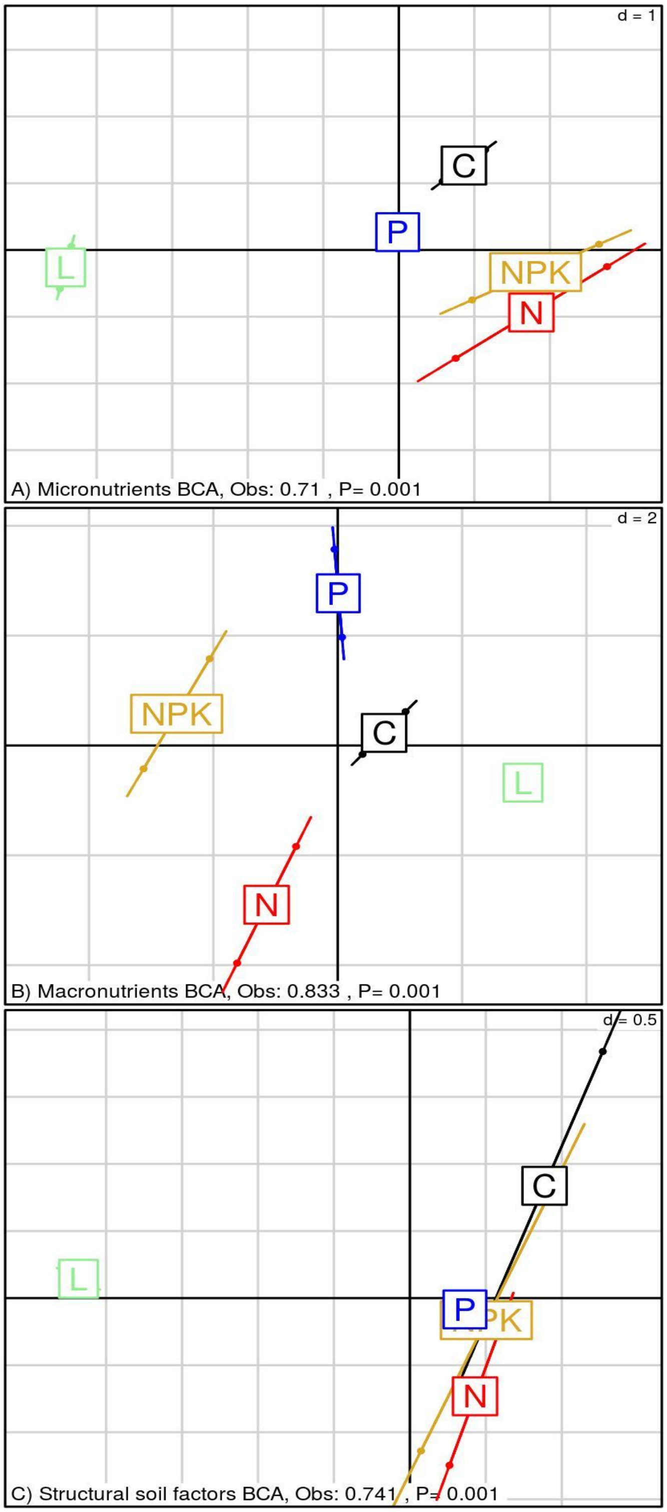

Supplementary Figure S4. Between-Class Analysis (BCA) of the soil factor subsets (A) micronutrients, B) macronutrients, C) structural) over the long-term control (C), liming (L), nitrogen (N), nitrogen-potassium-phosphorus (NPK) and phosphorus (P) treatments of the Ossekampen experiment are presented. Significance of groups was assessed by Monte-Carlo tests.

Supplementary Methods

Diversity and evenness calculations. Renyi diversities and evenness were calculated and visualized from the fungal (n=20), bacterial (n=27) datasets and the plant species (n=10) frequency dataset using the "BiodiversityR" R package. Plant richness was measured during sampling as the total number of species present. To obtain alpha diversity indices for the sequenced datasets, the bacterial and fungal OTU tables were rarified to the size of the smallest sample in each dataset. For the bacterial and the fungal samples, Renyi diversity indices at alpha = 1 (Shannon) and alpha = 2 (Inverse Simpson) were kept for group testing.

STAMP analysis. The fungal (n=20) and bacterial (n=27) datasets were agglomerated at the taxonomic rank of Phylum for the STAMP[1] analysis. Missing taxonomy information in the 18S rRNA dataset was resolved as follows: 1) if an entry was blank with classified entries before and after, then the blank was replaced with a copy of the entry after (eg. "Eukaryota", "", "Fungi", became "Eukaryota", "Fungi", "Fungi"), 2) if an entry was blank and the previous or following entry was "unclassified", it was replaced with "unclassified" (eg. "Eukaryota", "Fungi", "", "unclassified" became "Eukaryota", "Fungi", "unclassified", "unclassified") and 3) if an entry was blank and the previous entry was "Fungi" and the next entry was "fungal", the blank space was replaced with "Fungi." In addition, for the 18S rRNA dataset, ambiguous classifications (e.g. "environmental" and "unknown") were replaced with "unclassified". Unclassified reads were removed; then, the ANOVA statistical test was selected with a Tukey-Kramer post-hoc test (CI= 95%). A Benjamini-Hochberg FDR multiple test correction was applied.

Treatment effects on plant, bacterial and fungal communities and soil factor profiles. Between-Class Analysis (BCA) selects the orthogonal axis that maximizes between-group variance and measures the amount of variance restricted to the grouping factor as a percentage of the inertia captured through the new axis over the total inertia[2]. This allowed us to assess the amount of variability that could be explained by treatment for each community. The bacterial and fungal OTU abundances were summarized at the Phylum and Genus, or Phylum and Class levels, respectively. The plant species frequencies and the bacterial and fungal abundances were converted to relative abundances (contingency tables), and the soil factors were normalized and scaled to unit variance.

Between-component analyses. Co-inertia analysis is a multivariate method that identifies the common structure in two tables related by the same samples[3]. We chose this analysis because it can tolerate a high variable-to-sample ratio. Furthermore, we leveraged the imposed structure of the long-term fertilizer treatments. The co-inertia of two ordinations is the sum of squares of the co-variances of the variables in each table; thus, co-varying variables can be identified through correlation with the co-inertia axis[3]. Here we could simultaneously identify, for example, the taxonomic groups from two communities that contributed to treatment groupings in the factor map. The degree of multivariate co-variance between two ordinations is summarized within an array correlation and co-variance coefficient, the RV value, which is a measure of the global similarity.

References

1. Parks, D. H., Tyson, G. W., Hugenholtz, P. & Beiko, R. G. Stamp: statistical analysis of taxonomic and functional profiles. *Bioinformatics* **30**, 3123–3124 (2014).
2. Dray, S. & Dufour, A. B. The ade4 package: implementing the duality diagram for ecologists. *J. Statist. Software*, ***22***:1-20 (2007).

3. Dray, S., Chessel, D. & Thioulouse, J. Co-inertia analysis and the linking of ecological tables. *Ecology* **84**: 3078-3089 (2003).
